# Experimental exchange of paralogous domains in the MLH family provides evidence of sub-functionalization after gene duplication

**DOI:** 10.1093/g3journal/jkab111

**Published:** 2021-04-19

**Authors:** Christopher M Furman, Ryan Elbashir, Gianno Pannafino, Nathan L Clark, Eric Alani

**Affiliations:** Department of Molecular Biology and Genetics, Cornell University, Ithaca, NY 14853-2703, USA; Department of Molecular Biology and Genetics, Cornell University, Ithaca, NY 14853-2703, USA; Department of Molecular Biology and Genetics, Cornell University, Ithaca, NY 14853-2703, USA; Department of Human Genetics, University of Utah, Salt Lake City, UT 84132, USA; Department of Molecular Biology and Genetics, Cornell University, Ithaca, NY 14853-2703, USA

**Keywords:** gene duplication, gene specification, MLH proteins, mismatch repair, meiotic crossing over, *Saccharomyces cerevisiae*

## Abstract

Baker’s yeast contains a large number of duplicated genes; some function redundantly, whereas others have more specialized roles. We used the MLH family of DNA mismatch repair (MMR) proteins as a model to better understand the steps that lead to gene specialization following a gene duplication event. We focused on two highly conserved yeast MLH proteins, Pms1 and Mlh3, with Pms1 having a major role in the repair of misincorporation events during DNA replication and Mlh3 acting to resolve recombination intermediates in meiosis to form crossovers. The baker’s yeast Mlh3 and Pms1 proteins are significantly diverged (19% overall identity), suggesting that an extensive number of evolutionary steps, some major, others involving subtle refinements, took place to diversify the MLH proteins. Using phylogenetic and molecular approaches, we provide evidence that all three domains (N-terminal ATP binding, linker, C-terminal endonuclease/MLH interaction) in the MLH protein family are critical for conferring pathway specificity. Importantly, *mlh3* alleles in the ATP binding and endonuclease domains improved MMR functions in strains lacking the Pms1 protein and did not disrupt Mlh3 meiotic functions. This ability for *mlh3* alleles to complement the loss of Pms1 suggests that an ancestral Pms1/Mlh3 protein was capable of performing both MMR and crossover functions. Our strategy for analyzing MLH pathway specificity provides an approach to understand how paralogs have evolved to support distinct cellular processes.

## Introduction

Gene duplications, occurring through events such as polyploidization or unequal crossing over, can create new gene families that play important roles in adaptive evolution (Ohno 1970; Hughes 1994). In baker’s yeast, there are many examples of gene duplications that result in paralogs that appear to function redundantly or have specialized roles (Wolf and Shields 1997). Various models have been proposed to explain how novel protein functions have evolved after gene duplication. Hughes (1994) proposed that an ancestral gene initially existed prior to gene duplication that was capable of performing multiple functions. Following duplication, the two genes were subjected to purifying selection, leading to specialization (Hughes 1994). In another model, Force *et al.* (1999) proposed that neutral degenerative mutations can occur in each duplicated gene that complement each other [Duplication-Degeneration-Complementation (DDC) model], providing an opportunity for sub-functionalization. DDC has been proposed to resolve conflicts in cases where a single gene performing multiple functions acquires mutations that optimizes one function but negatively impacts others (Hittinger and Carroll 2007). The models outlined above can be challenging to test for an ancient duplication event, because the derived paralogs have diverged significantly in their amino acid sequences. In this study, we focused on the outcome of ancient gene duplication events that diversified the functions of the baker’s yeast MLH family of mismatch repair (MMR) genes. These events predated the whole-genome duplication event that took place ∼100 million years ago (Wolfe and Shields 1997; Marcet-Houben and Gabaldón 2015). First, we describe the MLH family proteins and their roles in MMR and meiotic crossing over, and then present our approach to alter their functions.

MMR is a highly conserved mechanism that reduces the genome mutation rate through the action of MutS homolog (MSH) proteins that bind to base–base and insertion/deletion mismatches that form as the result of DNA replication errors (Supplementary Figure S1, A and B). MSH recognition of mismatches results in the recruitment of the Mlh1-Pms1/PMS2 (also described as MutLα) endonuclease, which is activated through interactions with MSH proteins and the DNA replication processivity factor PCNA to nick the newly replicated daughter strand, leading to excision and resynthesis steps that maintain the original template information (Tran *et al.* 1997; Erdeniz *et al.* 2005; Kadyrov *et al.* 2006; Pluciennik *et al.* 2010; Goellner *et al.* 2015; Kawasoe *et al.* 2016).

Genetic and biochemical analyses in eukaryotic systems have identified MSH and MLH factors that have evolved new functions (Culligan 2000; Shell *et al.* 2007; Pyatnitskaya *et al.* 2019; Furman *et al.* 2021). For example, in baker’s yeast, the MSH family members Msh2-Msh6 and Msh2-Msh3 act in the repair of different subsets of mismatches, and Msh4-Msh5 promotes crossover formation in meiosis (Reenan and Kolodner 1992; Kunkel and Erie 2015; Manhart and Alani 2016). For the MLH family, baker’s yeast Mlh1-Pms1 plays a major role in MMR, whereas Mlh1-Mlh2 and Mlh1-Mlh3 display minor and more specialized roles in MMR but important roles in meiotic recombination. In meiosis, Mlh1-Mlh2 acts to regulate gene conversion tract length and Mlh1-Mlh3 acts in the biased cleavage of double-Holliday junctions (dHJs) to form crossovers ( Supplementary Figure S1, C and D; Flores-Rozas and Kolodner 1998; Harfe *et al.* 2000; Abdullah *et al.* 2004; Cotton *et al.* 2010; Zakharyevich et al. 2012; Romanova and Crouse 2013; Campbell *et al.* 2014; Manhart and Alani 2016; Duroc *et al.* 2017).

How does Mlh1-Mlh3 function in meiosis to facilitate crossover (CO) formation? Crossing over of parental homologs during meiotic prophase facilitates their segregation during the Meiosis I division. In the absence of at least one crossover event per homolog pair, nondisjunction events occur at high frequency, leading to the formation of aneuploid gametes (Manhart and Alani 2016). In baker’s yeast, the meiotic crossover pathway is initiated by the Spo11 complex, which catalyzes 150–200 DNA double-strand breaks (DSBs) genome wide (Keeney *et al.* 1997; Robine *et al.* 2007). These DSBs are resected in a 5ʹ to 3ʹ direction to form 3ʹ single-stranded tails that invade the homologous template to create a D-loop intermediate. In the major crossover pathway, the D-loop is further stabilized by ZMM proteins such as Msh4-Msh5 and Zip3 to enable DNA repair synthesis and branch migration, ultimately forming a dHJ intermediate that is asymmetrically cleaved in an Mlh1-Mlh3 and Exo1-dependent step to yield primarily crossover products (Zakharyevich et al. 2012; Manhart and Alani 2016). Recent work suggests that Mlh1-Mlh3 endonuclease functions are directed toward newly replicated DNA formed during the creation of the dHJ intermediate in a mechanism analogous to that proposed for the Mlh1-Pms1 nuclease during MMR (Moens *et al.* 2002; Abdullah *et al.* 2004; Kolas *et al.* 2005; Manhart *et al.* 2017; Marsolier-Kergoat *et al.* 2018). This is thought to be accomplished by Mlh1-Mlh3 being recruited to recombination intermediates through interactions with specific meiotic factors and the DNA polymerase processivity factor PCNA (Moens *et al.* 2002; Kolas *et al.* 2005; Cannavo *et al.* 2020; Kulkarni *et al.* 2020; Sanchez *et al.* 2020).

We asked if regions of Pms1 and Mlh3 could be identified that confer their functional specificities and if this information could be used to alter Mlh3 to make it a more robust MMR factor. Our work is based on a phylogenetic analysis indicating that Mlh1 homologs initially diverged from an MLH ancestor, followed by a splitting into Pms1 and Mlh3 sister groups. As described below, these efforts provided evidence that all three functional domains of the MLH proteins (N-terminal ATP binding, linker, C-terminal endonuclease/MLH interaction) have evolved for gene specialization. Importantly, a small number of mutations were created in *MLH3* that expanded its MMR specificity without disrupting its role in meiotic crossing over. This combination of approaches provides a strategy to understand how organisms have evolved paralog complexes with distinct cellular functions.

## Materials and methods

### Media

*Saccharomyces cerevisiae* SK1 and S288c strains were grown at 30°C in either yeast extract-peptone-dextrose media or minimal selective media (SC; Rose *et al.* 1990). When required, geneticin (Invitrogen, San Diego) was added at 200 µg/ml (Goldstein and McCusker 1999), and sporulation plates were prepared as described (Detloff *et al.* 1991).

### Plasmids

Plasmid and strain background derivation for the relevant MMR genes are listed in Supplementary Table S1. Plasmid constructs built by Gibson cloning were resub-cloned into the backbones of expression vectors. The DNA sequence of the open reading frame (including 300 bp upstream and 150 bp downstream) of constructs was confirmed by Sanger DNA sequencing (Cornell BioResource Center). Oligonucleotides and sequences of plasmids are available upon request.

Mlh3/Pms1 chimera integration vectors containing *MLH3_SK1_* and *PMS1_SK1_* sequences were derived from pEAI254, a 7.8 kb *MLH3_SK1_*::*KanMX* integrating vector (Al-Sweel *et al.* 2017), and pEAA238, a 9.1 kb *PMS1_SK1_ ARS-CEN HIS3* vector, respectively (Supplementary Figure S2). The Block mutation vectors were derived from pEAM168, a 10.7 kb *MLH3_SK1_*::*KanMX 2µ* vector, fragments of which were isolated to integrate *mlh3* alleles (Supplementary Figure S2; Supplementary Table S1). The *MLH3* and *PMS1* boundaries in the chimera plasmids were chosen based on structural and homology model analyses of MLH proteins that yielded three distinct regions; an ATP-binding domain, a linker region, and C-terminal endonuclease/MLH interaction domain (Supplementary Figure S3; Arana *et al.* 2010; Gueneau *et al.* 2013; Mlh3 homology model in Al-Sweel *et al.* 2017). The chimera plasmids were constructed by linking different domains of PCR-amplified *MLH3* and *PMS1* DNA sequences using NEB HiFi DNA Assembly cloning (New England Biolabs, Ipswich, MA, USA). For Mlh3, these domains were: ATP binding, aa 1 to 375; linker, 376 to 488; C-terminal endonuclease, 489 to 715. For Pms1, the domains were: ATP binding, aa 1 to 361; linker, 362 to 638; C-terminal endonuclease, 639 to 877 (SK1). The chimera constructs were assigned the following abbreviations: PMM [Pms1(1-361)-Mlh3(376-488)-Mlh3(489-715)]; MMP [Mlh3(1-375)-Mlh3(376-488)-Pms1 (639-877)]; PPM [Pms1(1-361)-Pms1(362-638)-Mlh3(489-715)]; MPP [Mlh3(1-375)-Pms1(362-638)-Pms1(639-877)]; PMP [Pms1(1-361)-Mlh3(376-488)-Pms1(639-877)]; MPM [Mlh3(1-375)-Pms1(362-638)-Mlh3(489-715)].

*mlh3* Block mutations were constructed using NEB HiFi DNA Assembly cloning and/or Q5 site-directed mutagenesis kits (New England Biolabs, Ipswich, MA, USA). The integration plasmids were digested with *Bam*HI and *Sal*I prior to transformation into EAY3255 using methods described by Gietz *et al.* (1995). At least three independent transformants for each genotype were made and genotyped by PCR (presence of specific alleles also confirmed by DNA sequencing) using primers that map outside of the restriction sites used for integration.

*MLH3/PMS1* chimera and Block mutation alleles (SK1 background) were also expressed from the native *MLH3* promoter on *2µ LEU2* plasmids or on *ARS-CEN HIS3* plasmids. pEAI254 (*MLH3_SK1_::KanMX*), pEAM168 (*MLH3_SK1_::KanMX, 2µ*), pEAM65 (*MLH3_SK1_::LEU2, 2µ*), and pEAA636 (*MLH3, HIS3, ARS-CEN)* were the parental plasmids for these constructs. These plasmids were tested for complementation of MMR defects in the S288c strain EAY3097 (relevant genotype *pms1Δ*, *lys2::insE-A_14_*).

### Strains

The SK1 strains EAY3252, EAY3255, and EAY3486 and indicated derivatives were used to measure Mlh3-dependent meiotic crossing over and MMR functions (Supplementary Table S2). EAY3255 and derivatives contain the *lys2::insE-A_14_* allele to measure mutation rate (Tran *et al.* 1997). EAY3252/EAY3486 (*wild type*), EAY3255/EAY3486 (*mlh3Δ*) and EAY3255::*mlh3* alleles/EAY3486 diploids contain spore-autonomous fluorescence markers to measure meiotic crossing over in the *CEN8-THR1* interval (Supplementary Figure S1D; Thacker *et al.* 2011). The S288c strain EAY3097 (relevant genotype *pms1Δ*, *lys2::insE-A_14_*) was used to determine if *mlh3* alleles (SK1 background) expressed on *2µ* and *ARS-CEN* vectors could restore MMR functions in *pms1Δ* strains (Table 2). The S288c strain EAY4595 (relevant genotype *mlh3*Δ) was used to show that the SK1 derived *MLH3* gene fully complemented the MMR defects seen in the strain.

### lys2-A_14_ reversion assay

The haploid strains described in Tables 1 and 2 and Supplementary Table S2 were analyzed for MMR functions using the *lys2-A_14_* reversion assay (Supplementary Figure S1B; Tran *et al.* 1997). *ARS-CEN* and *2 μ* vectors were maintained by growing strains in minimal leucine dropout media. Rates of *lys2::insE-A_14_* reversion were calculated as μ = f/ln(N·μ) where f is the reversion frequency and N is the total number of revertant in the culture (Tran *et al.* 1997). For each strain, 15–44 independent cultures, obtained from two to four independent transformants, were assayed on at least two different days to prevent batch effects, and 95% confidence intervals were determined as described by Dixon and Massey (1969). The Mann–Whitney U test was used to calculate median reversion rates (Drake 1991).

**Table 1:** Functional analysis of *MLH3/PMS1* chimera and *mlh3* alleles in MMR and meiotic crossing over (CO)

	MMR			Meiotic CO		
Genotype	Rate × 10^−6^ (n)	95% CI. × 10^−6^	Relative to WT	% tetratype (*n*)	Phenotype	
					MMR	CO
*MLH3*	1.03 (42)	0.81–1.39	1	37.1 (1023)	+	+
*mlh3Δ*	6.24 (39)	4.53–8.51	6.05	18.3 (1239)	−	−
*MLH3-PMS1* chimeras						
* PPP (PMS1)*	8.29 (15)	2.85–22.2	8.1	17.8 (549)	−	−
* PMM*	15.6 (15)	3.29–29.0	15.1	19.2 (530)	−	−
* MMP*	19.1 (15)	12.9–25.4	18.6*	21.4 (524)	− −	−
* PPM*	15.9 (15)	11.8–128	15.4*	19.8 (824)	− −	−
* MPP*	15.4 (15)	12.4–32.3	14.9*	22.3 (837)	− −	−
* PMP*	17.2 (15)	13.1–18.2	16.7*	20.7 (1,287)	− −	−
* MPM*	8.75 (15)	5.11–11.4	8.49	21.4 (1,196)	−	−
*mlh3* Block mutations						
* *Block 1, ATP binding						
* mlh3-K17T, A20Q, S24D, R30K, Q34D*	2.26 (15)	1.18–3.78	2.20	35.6 (513)	+/−	+
* *Block 2, Mlh1 interaction						
* mlh3-Y493M, N497G, V499F, D500N, K502G*	3.71 (15)	2.04–4.98	3.60	29.1 (769)	+/−	+/−
* mlh3-D500N*	1.73 (15)	1.33–6.98	1.68	33.1 (801)	+/−	+
* mlh3-K502G*	2.84 (15)	2.13–4.76	2.76	19.1 (761)	+/−	−
* *Block 3, Endonuclease motif						
* mlh3-R530K*	2.38 (15)	1.61–3.28	2.31	25.3 (771)	+/−	+/−
* mlh3-R532N*	1.25 (15)	0.91–2.72	1.21	29.5 (774)	+	+/−
* mlh3-R530K, R532N*	4.49 (15)	3.64–7.24	4.36	20.4 (509)	−	−
* *Block 4, PCNA interaction motif						
* mlh3-PIP1*	3.49 (15)	1.96–4.35	3.39	30.9 (742)	−	+/−
* mlh3-PIP2*	0.567 (15)	0.41–0.75	0.55	37.1 (792)	+	+
* *Block 5, Helix 2						
* mlh3-V660K, N666A, F676I, D678K*	4.44 (15)	2.45–12.8	4.32	18.2 (760)	−	−
* mlh3-D678K*	0.831 (15)	0.57–1.18	0.81	32.1 (772)	+	+/−
* *Block 6, Helix1						
* mlh3-C695L, F699W, A702P*,	3.38 (15)	2.37–4.76	3.28	19.5 (527)	+/−	−
* S707T, V709R, P710H*						
* *Mutant combinations						
* *Block 1 (*ATP binding*), Block 4 (*PIP2*)	2.56 (15)	1.95–4.05	2.48	31.6 (509)	+/−	+/−
* *Block 5 (*Helix 2*), Block 6 (*Helix 1*)	4.11 (15)	2.40–5.28	3.99	17.9 (514)	+/−	−
S288C background						
* wild type + empty vector*	0.28 (15)	0.19–0.47	**1.00**		+	
* mlh3Δ + empty vector*	1.61 (15)	1.37–2.08	5.90		−	
* mlh3Δ + pMLH3_SK1_ ARS-CEN*	0.27 (15)	0.18–0.39	0.98		+	
* mlh3Δ + pMLH3_SK1_-2µ*	223 (15)	209–291	81.8*		− −	

The indicated *mlh3* Block alleles and chimeras (Supplementary Table S1; *Materials and Methods*) were integrated into the *MLH3* locus in the SK1 strain background and tested for DNA MMR functions using the *lys2-A_14_* reversion assay with 95% CI (confidence interval) presented, and for meiotic crossover functions using a spore-autonomous assay that measures genetic map distances in the *CEN8-THR1* interval on Chromosome VIII (*Materials and Methods*; Supplementary Table S2). For the S288c background experiments, EAY1269 (*wild type*, S288c background) was transformed with pRS415 (empty vector) and EAY4595 (*mlh3Δ*) was transformed with pRS415 (empty vector), *MLH3_SK1_-ARS-CEN* (pEAA566), and *MLH3_SK1_-2 micron* (pEAM65; Supplementary Table S1)*. n* represents the number of independent measurements from at least two transformants. WT, wild type. +, indistinguishable from *MLH3* as measured by 95% CI or Chi-Squared (*P* < 0.0001 for % tetratype). −, indistinguishable from *mlh3Δ* as measured by 95% CI or Chi-Squared (*P* < 0.0001). +/− distinguishable from both *MLH3* and *mlh3Δ* as measured by 95% CI or Chi-Squared (*P* < 0.05). *Mutation rate higher than *mlh3Δ*; illustrated as a − − phenotype. Note that while expression of *MLH3* on an *ARS-CEN* vector fully complements the mutator phenotype seen in an *mlh3Δ* strain, expression of *MLH3* on a *2µ* high copy vector confers a mild mutator phenotype, most likely by sequestering Mlh1 from interacting with Pms1 (Nishant *et al.* 2008).

### Spore-autonomous fluorescence assay to measure percent tetratype

Diploids in the EAY3252/EAY3486 background (Supplementary Table S2) were used for analysis of meiotic crossing over phenotypes. These diploids contain a spore-autonomous fluorescent protein marker (CFP) linked to *THR1* of Chromosome VIII and an RFP marker linked to *CEN8* in the second copy (Supplementary Figure S1D; Thacker *et al.* 2011). Diploids were selected by mating parental and derived EAY3252 and EAY3486 strains on media lacking tryptophan and leucine and maintained as stable strains. Fluorescence microscopy was used to quantify parental ditypes and tetratypes resulting from single crossover events. Sporulation plates were prepared as described by Detloff *et al.* (1991) and incubations were performed at 30°C. Spores were treated with 0.5% NP40 and sonicated for 5–10 s before analysis using a Zeiss Axioimager.M2. 250–1000 tetrads for each *mlh3* allele were counted to determine % tetratype [# tetratypes/(tetratypes + parental ditypes)]. Two to three independent transformants were measured per allele on at least two different days to prevent batch effects. In this assay, wild-type SK1 *S. cerevisiae* strains gave single crossover events at 37.1% frequency, whereas *mlh3* null strains gave single crossover events at 18.3% frequency (Table 1). A Pearson’s Chi-Squared contingency test (http://vassarstats.net/) was used to test statistical significance to classify each allele as exhibiting a wild type, intermediate, or null phenotype. We applied a Benjamini–Hochberg correction at a 5% false discovery rate to minimize α inflation due to multiple comparisons (30 comparisons, with a *P* < 0.0183 cutoff for significance; Supplementary Table S3).

### Evolutionary analysis of fungal Mlh1, Mlh2, Mlh3, and Pms1 proteins

**Figure 1:**
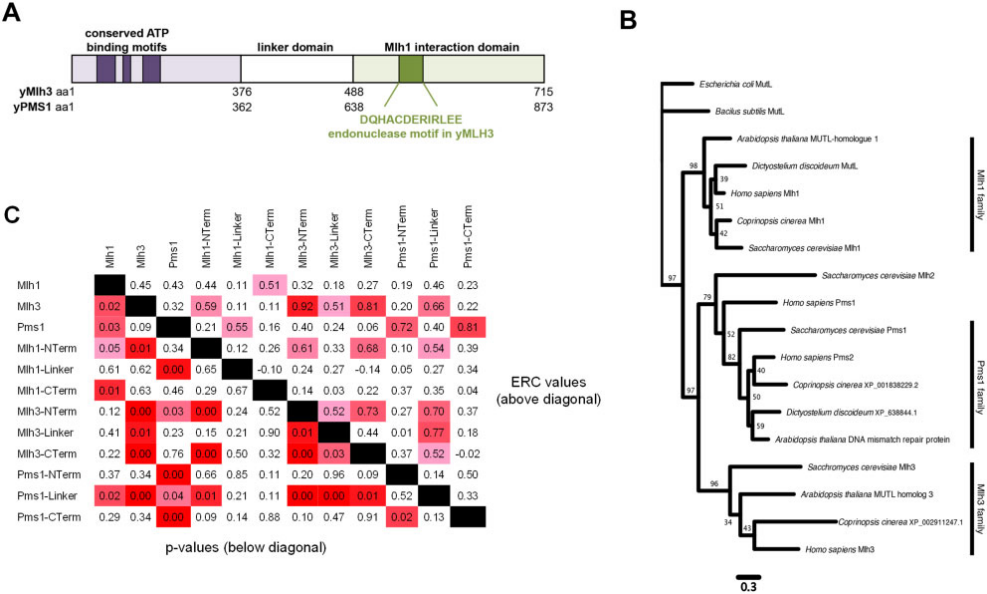
Phylogenetic and ERC analysis of MLH proteins. (A) Cartoon depictions of the Mlh1-Mlh3 and Mlh1-Pms1 complexes, with the N-terminal ATP binding and C-terminal endonuclease/Mlh1 interaction domains separated by intrinsically disordered linker domains. The amino acid locations of the domains in yeast Mlh3 and Pms1 are shown. (B) Phylogenetic analysis of divergent eukaryotes indicates that *MLH* gene duplications occurred in early eukaryote history before the divergence of the lineages leading to plants, fungi, animals, and amoebozoa. The scale bar indicates the number of changes per amino acid site, and bootstrap support values out of 100 are found near each node. *Escherichia coli* MutS and *B. subtilis* MutL were used to root the tree. The branching pattern supports the earliest divergence of the Mlh1 family followed by splits to form the Mlh2, Mlh3, and Pms1 families. Uniprot, NCBI, and RefSeq identifications for the genes used to make the tree can be found in Supplementary Table S4. (C) ERC is elevated between domains of MLH proteins. This pairwise matrix shows all comparisons between the full-length Mlh1, Mlh3, and Pms1 proteins and their ATP binding (abbreviated as Nterm), linker, and endonuclease/MLH interaction (abbreviated as Cterm) domains defined in (A). The domain boundaries of the 18 fungal species were obtained from phylogenetic alignments with the *S. cerevisiae* proteins. ERC values are above the diagonal and empirical *P*-values are below. The colors of ERC cells range from pink at values of 0.5 to red at 1.0. *P*-value cells are pink at 0.05 and become red as they approach zero.

The inferred amino acid sequences of Mlh1, Mlh3, Pms1, and Pms2 were located by BLAST against the gene annotations of *Homo sapiens*, *Coprinopsis cinerea*, *Arabidopsis thaliana, S. cerevisiae*, and *Dictyostelium discoideum* (Figure 1B; Supplementary Table S4). *Escherichia coli* MutS and *Bacillus subtilis* MutL were used to potentially root the tree. To decipher relationships between MutL homologs, amino acid sequences from the entire open reading frames of the Mlh1, Mlh2, Pms1, and Mlh3 proteins were aligned in SeaView (Version 4, Gouy *et al.* 2010) using the MUSCLE program (Version 3.8.31, Edgar 2004). Since these sequences were very divergent, the alignment was aggressively trimmed by eye to only 216 columns to maintain the most confidently aligned and conserved regions. Phylogenetic analyses were performed with PhyML (Guindon and Gascuel 2003) in SeaView using model-provided amino acid equilibrium frequencies, optimized across site rate variations, and bootstrapping with 100 replicates.

The inferred amino acid sequences of Mlh1, Mlh3, and Pms1 were also taken from the complete genome sequences of 34 *Ascomycetes* fungal species (GenBank, https://www.ncbi.nlm.nih.gov/genbank/; Supplementary Figure S4A). *Escherichia coli* MutS and *B. subtilis* MutL were used to potentially root the tree. Twenty-nine Mlh3, 34 Pms1, 18 Mlh2, and 33 Mlh1 proteins were analyzed in the tree analysis. A gene missing from a particular species does not necessarily indicate gene loss in that species but could reflect incomplete genome sequencing or other bioinformatic difficulty in locating the orthologous sequence. To decipher relationships between MutL homologs, amino acid sequences from the entire open reading frames of the Mlh1, Mlh2, Pms1, and Mlh3 proteins were aligned in SeaView (Gouy *et al*. 2010) using the Clustal Omega program (Edgar 2004). Phylogenetic analyses were performed with PhyML (Guindon and Gascuel 2003) in SeaView using model-provided amino acid equilibrium frequencies, optimized across site rate variations, and bootstrapping with 100 replicates. A second phylogenetic analysis was performed on a subset of more conserved alignment columns as selected by the Gblocks program (Supplementary Figure S4B; Castresana 2000). Specifically, GBlocks was used to select more conserved blocks of the alignment by excluding columns that include or flank gap characters and that have high divergence as determined by the threshold in GBlocks. This reduced the alignment from 1509 to 212 highly conserved and more confidently aligned positions. A phylogenetic tree was then inferred using PhyML under the same parameters and model as before. Phylogenetic tree images were created and annotated using interactive Tree of Life v.4 online tool (Letunic and Bork 2019).

### Evolutionary rate covariation analysis

The 18 species included for the evolutionary rate covariation (ERC) analysis were: *S. cerevisiae*, *Saccharomyces paradoxus*, *Saccharomyces mikatae*, *Saccharomyces bayanus*, *Naumovozyma castellii*, *Candida glabrata*, *Vanderwaltozyma polysporus*, *Lachancea kluyveri*, *Lachancea thermotolerans*, *Lachancea waltii*, *Kluyveromyces lactis*, *Eremothecium gossypii*, *C. tropicalis*, *C. albicans*, *C. dubliniensis*, *Candida lusitaniae*, *C. guilliermondii*, *and Debaryomyces hansenii*. These 18 *Ascomycetes* fungal species were chosen for ERC to avoid very long branches, which can confound the correlations by producing outlier values. Multiple species alignments for Mlh1, Mlh3, and Pms1 were subdivided into N-terminal, Linker, and C-terminal domains so that they could be analyzed separately (Figure 1A). ERC values between a given pair of proteins or domains were calculated as initially described in Clark *et al.* (2012, 2013) with modifications made to improve the relative rate normalization (Figure 1C; Partha *et al.* 2019). Briefly, the branch lengths for each domain or protein tree were first normalized into relative evolutionary rates (RERs) using the RERconverge package (Kowalczyk *et al.* 2019). Normalization vectors were estimated from the branch lengths of 4458 proteins plus the three MLH domains in Mlh1, Pms1, and Mlh3 (9 in total). The resulting RERs were used to calculate the ERC values between all pairwise comparisons of the three domains in Mlh1, Mlh3, and Pms1. ERC values were calculated as the Pearson correlation coefficients between each pair of domains with extreme values controlled by limiting the two most extreme positive and negative branch RERs to the third most extreme value (*i.e.*, Winsorization of the two most extreme outliers).

### Using multi-Harmony to identify sites for mutagenesis in MLH3

**Figure 2:**
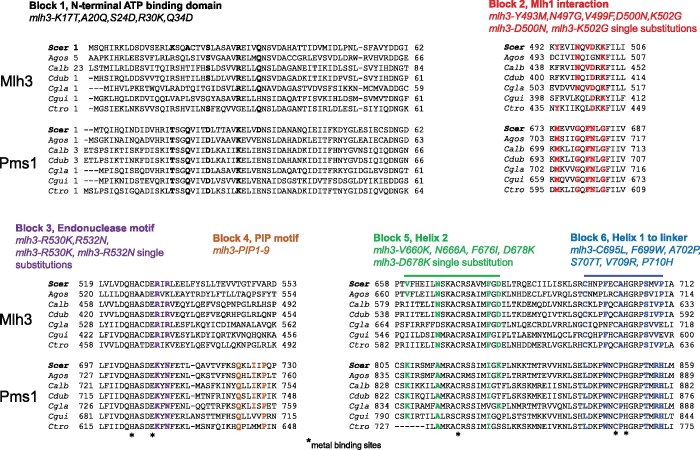
Mutations made in *MLH3* to revert back to conserved *PMS1* sequences. Six blocks of mutations were made in Mlh3; Block 1-ATP binding, Block 2-Mlh1 interaction, and Blocks 3 to 6-Endonuclease/PCNA interaction. The multiple amino acid substitutions are shown for each block as well as single substitutions that were made in each region. For Blocks 1, 2, 5, and 6, Multi-Relief and Sequence-Harmony algorithms were used to identify functionally specific residues in Pms1 (*Materials and Methods*; adapted from Furman *et al.* 2021). Briefly, Mlh3 and Pms1 amino acid sequences from 34 different fungal species were aligned and presented in multi-Harmony. Shown are alignments of the regions showing seven fungal species (*S. cerevisiae-Scer*, *Ashbya gossypii-Agos*, *Candida albicans-Calb*, *Candida dubliniensis-Cdub*, *Candida glabrata-Cgla*, *Candida guilliermondii-Cgui*, *Candida tropicalis-Ctro*). The more complete lists of species alignments are shown in Supplementary Figure S4 legend. The N and C-terminal domains of Mlh3 were mapped onto the 3D structure of Mlh1-Pms1, and four amino acid clusters were identified for substitution analysis (Manning *et al.* 2008; Brandt *et al.* 2010). Block 3 spans the endonuclease motif found in Pms1 and Mlh3 (Supplementary Figure S3D). Block 4 contains the QXLXXP motif important for interactions with PCNA (PIP), which is highly conserved in the Pms1 sequences (>94% identity; Genschel *et al.* 2017) but is absent in Mlh3 sequences. Nine PIP mutations were made as shown in Supplementary Figure S3D and Tables 1 and 2. The asterisks indicate highly conserved metal-binding residues (H703, E707, C817, C848, and H850) in yeast Pms1, which form the endonuclease active site (Gueneau *et al.* 2013).

To identify specificity determining residues in Mlh3 and Pms1, alignments of Pms1 and Mlh3 fungal species (34 for Pms1; 29 for Mlh3) were used for multi-Harmony analysis (Figure 2; Supplementary Figure S5, A–D; Brandt *et al.* 2010). Using multi-Harmony, amino acid positions significantly different between the two groups of sequences were identified. An identity cutoff of a combination of a score for multi-relief greater than 0.8 and a score less than 0.5 for sequence harmony was used to reduce the number of interesting amino acids resulting in a set of group-specific positions. Once identified, the amino acids were mapped onto a homology model of Mlh3 from Al-Sweel *et al.* (2017) using PyMOL to verify and group amino acids qualitatively in close physical proximity. This analysis led to the identification of Blocks 1, 2, 5, and 6 (Figure 2; Supplementary Figure S5, A–D). Blocks 3 and 4 were identified for analysis based on the identification of endonuclease and PCNA-binding motifs found in *B. subtilis* MutL and a subset of MLH homologs (Pillon *et al.* 2011; Supplementary Figure S3D).

**Table 2:** *mlh3-ATP binding and mlh3-PIP* mutations modestly rescue *pms1* null MMR defects

*pms1Δ* strain with indicated plasmids	Rate × 10^−7^ (*n*)	95% CI × 10^−7^	Relative to wild type
*PMS1, ARS-CEN*	2.99 (30)	2.11–3.85	**1**
*PMS1, 2µ*	22.4 (18)	15.1–63.5	7.50
*ARS-CEN (LEU2)*	20,300 (29)	12,900–28,700	6,800
*2µ*	18,900 (18)	15,900–21,300	6,320
*mlh3* Block mutations, *ARS-CEN*			
* MLH3*	14,700 (15)	8,620–19,700	4,910
* *Block 4 *(mlh3-PIP1)*	14,400 (15)	6,470–18,600	4,830
* *Block 4 *(mlh3-PIP2)*	11,300 (15)	7,890–21,200	3,790
*mlh3* Block mutations, *2µ*			
* *Block 1, ATP binding			
* mlh3-K17T, A20Q, S24D, R30K, Q34D*	9,720 (18)	6,360–11,300	3,250*
* *Block 2, Mlh1 interaction			
* mlh3-Y493M, N497G, V499F, D500N, K502G*	15,400 (18)	10,700–23,700	5,160
* mlh3-D500N*	10,900 (18)	9,540–16,100	3,630
* mlh3-K502G*	16,500 (18)	10,200–19,100	5,530
* *Block 3, Endonuclease motif			
* mlh3-R530K*	20,500 (18)	16,900–28,600	6,840
* mlh3-R532N*	15,700 (18)	10,600–21,600	5,240
* mlh3-R530K, R532N*	13,300 (18)	10,100–17,200	4,440
* *Block 4, PCNA interaction motif			
* MLH3* (GTFVAR)	13,200 (28)	8,280–20,600	4,430
* mlh3-PIP1* (QKLIIP)	13,700 (25)	7,940–17,200	4,590
* mlh3-PIP2* (QTFIAP)	6,300 (44)	4,120–7,470	2,110*
* mlh3-PIP3 (QTLIAP)*	8,520 (18)	6,950–17,600	2,850
* mlh3-PIP4* (GTFIAP)	13,800 (18)	7,390–22,400	4,610
* mlh3-PIP5* (QTFIAR)	6,890 (18)	4,250–9,890	2,300*
* mlh3-PIP6* (QTFVAP)	15,700 (18)	13,100–25,800	5,240
* mlh3-PIP7* (GTFVAP)	15,500 (18)	10,500–22,500	5,190
* mlh3-PIP8* (GTFIAR)	19,500 (18)	15,100–25,900	6,510
* mlh3-PIP9* (QTFVAR)	8,520 (18)	4,890–10,800	3,060*
* *Block 5, Helix 2			
* mlh3- V660K, N666A, F676I, D678K*	14,900 (18)	10,700–19,700	4,970
* mlh3-D678K*	14,700 (18)	11,900–22,200	4,920
* *Block 6, Helix 1			
* mlh3-C695L, F699W, A702P, S707T, V709R, P710H*	14,100 (18)	12,100–21,100	4,710
Double mutants			
* *Block 1, Block 4 (*PIP2*)	5,480 (18)	4,340-6,710	1,830*
* *Block 2 (*D500N)*, Block 4 *(PIP2*)	12,200 (18)	6,340–15,100	4,078
* *Block 2 (*K502G)*, Block 4 *(PIP2*)	11,900 (18)	6,830–19,100	3,970
* *Block 3 (*R530K)*, Block 4 *(PIP2*)	7,350 (18)	2,490–12,700	2,460*
* *Block 3 (*R532N)*, Block 4 *(PIP2*)	7,110 (18)	3,240–9,650	2,380*
* *Block 3 (*R530K, R532N)*, Block 4 *(PIP2*)	17,600 (18)	13,400–19,800	5,880
* *Block 4 *(PIP2*), Block 5 (*D678K*)	11,000 (18)	5,750–12,900	3,690*
* *Block 4 (*PIP2*), Block 5 (*Helix 2*)	18,700 (18)	12,200–30,900	6,240
* *Block 4 (*PIP2*), Block 6 (*Helix 1*)	20,600 (18)	14,400–27,900	6,904
* *Block 5 (*Helix 2*), Block 6 (*Helix 1*)	20,200 (18)	17,500–38,800	6,740
*MLH3-PMS1* chimeras*, 2µ*			
* MMM*	13,200 (28)	8,280–20,600	4,430
* PPP*	42.9 (18)	24.1–91.4	14.4
* PMM*	19,600 (18)	8,530–31,200	6,550
* MMP*	14,600 (18)	11,600–21,100	4,870
* PPM*	16,100 (18)	12,900–18,500	5,380
* MPP*	14,800 (18)	11,800–18,300	4,960
* PMP*	15,300 (18)	12,800–21,300	5,130
* MPM*	20,400 (18)	18,100–35,100	6,830

EAY3097 (*pms1Δ, S288c background*) was transformed with pJH481 (*PMS1_S288c_*, *ARS-CEN*), pEAM50 (*PMS1_S288c_*, *2µ*), and pRS415 (dummy vector; Supplementary Table S1) to analyze *PMS1*, *PMS1-2µ*, and *pms1Δ* genotypes, respectively. *mlh3* substitution alleles (Blocks 1–6, Figure 2; Supplementary Table S1) and *MLH3-PMS1* chimera constructs (Supplementary Table S1) were also transformed into EAY3097. *mlh3* alleles and chimeras were expressed from an *MLH3* promoter in *2µ* and *ARS-CEN* vectors as indicated. All strains were analyzed for mutation rate using the *lys2-A_14_* reversion assay as described in the *Materials and Methods* with the 95% confidence interval (CI) presented. *n* represents the number of independent measurements obtained from at least two transformants. *Indicates complementation of *pms1Δ* as measured by nonoverlap in 95% CI.

### Data availability

Strains and plasmids are available upon request. Supporting information contains all detailed descriptions of Supplemental files. All experiments presented (*lys2-A_14_* reversion, CO assays) were repeated on at least two separate days. The Supplementary figures and tables can be found in the GSA Figshare portal: https://doi.org/10.25387/g3.14367533.

## Results

### Phylogenetic analysis supports MLH gene duplication being an ancient event

MLH family proteins contain three structurally conserved regions; an N-terminal ATP-binding domain that facilitates conformational changes, an intrinsically disordered linker arm, and a C-terminal domain containing a region required for dimerization with other MLH proteins. Many MLH proteins also contain a functional endonuclease domain within the C-terminal region (Figure 1A; Supplementary Figure S3D; Ban and Yang 1998; Guarné *et al.* 2004; Arana *et al.* 2010; Gueneau *et al.* 2013). While showing an overall organizational similarity, the baker’s yeast Mlh1, Mlh2, Mlh3, and Pms1 proteins display significant amino acid divergence. For example, Mlh3 (715 amino acids in length) and Pms1 (873 amino acids) display limited (19%) amino acid identity over a 1010 amino acid gapped alignment (Supplementary Figure S6), with the greatest divergence seen in the linker domain (7.9% identity, with many gaps).

To better understand how the Mlh3 and Pms1 proteins diverged, we performed two phylogenetic tree analyses of homologs of the MLH paralogs. The first was performed with a diverse set of eukaryotic lineages (note that human Pms2 and Pms1 are homologs of fungal Pms1 and Mlh2, respectively), with the goal of estimating when *MLH* family gene duplication events occurred. As shown in Figure 1B, paralog branching patterns of highly diverged eukaryotes suggest first a split of the ancestral *MLH* gene to form the Mlh1 family and the ancestor of Mlh3/Pms1, followed by splits to form the Mlh2, Mlh3, and Pms1 families. These observations indicate that *MLH* gene duplications occurred early in eukaryote history, before the divergence of lineages leading to Plantae (plants), Opisthokonts (fungi/metazoa), and Amoebozoa. Note that although the MLH families were well resolved, the branching order of the taxonomic lineages was not well resolved within each family because of insufficient alignable sequence in the paralogs.

To better define the steps leading to the divergence of the MLH homologs, we performed the second phylogenetic tree analysis on Mlh1, Mlh2, Mlh3, and Pms1 homologs from 34 Ascomycete species (Supplementary Figure S4A; *Materials and Methods*; Clark *et al.* 2013). This analysis includes species both before and after the whole-genome duplication event (∼100 million years ago), which took place after the split of the yeast species into the *Kluyveromyces* and *Saccharomyces* clades. The identification of Mlh1, Mlh2, Mlh3, and Pms1 homologs in both clades confirms that Mlh1, Mlh2, Mlh3, and Pms1 predated the whole-genome duplication event (Wolfe and Shields 1997; Campbell *et al.* 2014). Similar to the analysis presented in Figure 1B, the Ascomycota analysis supports the divergence of Mlh1 (outgroup) first, followed by another split into Pms1, Mlh2, and Mlh3 as sister groups. While the trees are consistent with Mlh3, Mlh2, and Pms1 sharing a more recent common ancestor and function than with Mlh1, it is difficult to definitely conclude directionality because the events are ancient. To further scrutinize the inferred divergence order, we analyzed a select subset of more conserved and confidently aligned amino acid sites for Mlh1, Mlh3, and Pms1. Specifically, we used the GBlocks program to select more conserved blocks of the alignment lacking insertion and deletion events. The phylogenetic tree inferred with those 212 sites also showed Mlh1 diverging first, followed by Mlh3 and Pms1, and those relationships were supported by high approximate likelihood ratio test branch support values (Supplementary Figure S4B).

The data in Supplementary Figure S4 also indicate an acceleration of changes in the Mlh2 and Mlh3 clades; the continued faster rate likely reflects relaxed constraint, though neofunctionalization for Mlh2 (regulating gene conversion tracts) and Mlh3 (resolving recombination intermediates into crossovers) to act in meiotic recombination is an alternative explanation. It is difficult to make a definitive conclusion on this point because the events are ancient. Estimating selective pressure during that ancient divergence is challenging (*e.g.*, measuring *d*_N_/*d*_S_, the ratio of the number of nonsynonymous to synonymous substitutions per site), because outside of the genus *Saccharomyces* the synonymous sites are saturated. The data also suggest that Mlh1 remains under stronger constraint relative to Mlh3, which is curious because Mlh1 and Mlh3 are hypothesized to be coevolving as measured by ERC analysis (Clark *et al.* 2012). It is important to note that proteins with different average evolutionary rates can still have correlated changes in their rates over time, and that Mlh3, Pms1, and Mlh2, have all maintained their interaction with Mlh1, indicating that such interactions are likely to impose evolutionary constraints.

### ERC signals are seen between sets of domains for three MLH proteins

The phylogenetic analysis above, coupled with the previously defined functions of Mlh1-Pms1 and Mlh1-Mlh3 in MMR and meiotic crossing over, respectively, encouraged us to focus on what domains in Pms1 and Mlh3 were critical for conferring pathway specificity. ERC, which identifies protein pairs with correlated changes in evolutionary rate, has been used to make functional inferences (Clark *et al.* 2012, 2013). In general, ERC values between unrelated proteins do not show correlated rate changes, whereas protein pairs in shared pathways, complexes, and functions show positively correlated rates (Clark *et al.* 2012). ERC is calculated as the correlation coefficient between the phylogenetic branch-specific rates of one protein *vs* another. A value of one indicates perfect rate covariation and a value near zero represents little or no covariation. Previous studies had shown an elevated ERC for Mlh1 and Mlh3 with each other and with meiotic crossover proteins (Clark *et al.* 2013), but ERC values for Mlh1 and Mlh3 with the MMR-specific components Msh2 and Msh6 were not elevated, suggesting that the evolutionary forces relating to meiotic crossing over had a greater effect on Mlh1 and Mlh3 than MMR.

ERC has been used previously to compare rates between full-length protein sequences. In this study, we performed ERC analysis on the whole-length proteins as well as the ATP binding (N-terminal), linker, and endonuclease/MLH interaction (C-terminal) domains of Mlh1, Mlh3, and Pms1 using 18 yeast species (including *S. cerevisiae*; *Materials and Methods*; Clark *et al.* 2013). The purpose of this analysis was to determine if any one specific domain of the MLH proteins was showing ERC with another, or if multiple domains displayed such covariation as compared to the whole protein. This analysis involved the 12 × 12 matrix presented in Figure 1C. It is important to note that our new analysis showed slightly lower levels of ERC between Mlh1 and Mlh3 than previously reported (Clark *et al.* 2013). This difference is due to normalizing branch lengths more carefully, resulting in an improved way to compute RERs (*Materials and Methods*). With this new method, we see similarly significant ERC signals between Mlh1 and Mlh3, and Mlh1 and Pms1 (*P* < 0.05). Elevated ERC signals (*P* ≤ 0.03) were seen between the individual Mlh3 domains as well as between the three Mlh3 domains and the Pms1 linker. They were also seen between the Mlh1 N-terminal and the Mlh3 N-terminal, Mlh3 C-terminal, and Pms1 linker domains. Finally, an elevated ERC signal was seen between the Pms1 N-terminal and Pms1-C-terminal domains. These observations do not show a specific pattern of signals between specific domains of MLH proteins but are consistent with structural studies indicating that ATP-dependent conformational rearrangements involving the linker regions of the MLH proteins are important for the positioning of the two N-terminal MLH domains and bound DNA near the endonuclease active site in the C-terminus (Ban *et al.* 1999; Sacho *et al.* 2008; Pillon *et al.* 2010).

### Chimeric Mlh3/Pms1 proteins interfere with Mlh3-dependent MMR

Phylogenetic and ERC analyses indicating that Mlh3 and Pms1 are sister groups and that the three domains of MLH proteins covary encouraged us to perform domain swaps between Mlh3 and Pms1. These were performed to determine if we could alter Mlh3 and Pms1 functions by exchanging domains. We tested for altered specificities in MMR in *mlh3Δ* and *pms1Δ* strains using a highly sensitive *lys2-A_14_* reporter containing a centrally located homopolymeric run of 14 deoxyadenosine residues that result in a + 1-frameshift mutation (Supplementary Figure S1B; Tran *et al.* 1997). DNA slippage events that restore the reading frame, primarily the result of -1-frameshift mutations, confer reversion to Lys^+^ (*Materials and Methods*). We measured meiotic crossing over using a spore-autonomous fluorescence assay to measure percent tetratype at the *CEN8-THR1* interval (Supplementary Figure S1D; Thacker *et al.* 2011). Fluorescence microscopy was used to quantify parental ditypes and tetratypes resulting from single crossover events. Two red and two blue spores are detected in the absence of a crossover (parental ditype) between the RFP and CFP markers. For a single crossover (tetratype), a tetrad contains one red, one blue, one purple, and one nonfluorescent spore.

Six Mlh3/Pms1 chimeric proteins were constructed in which the N-terminal, linker, and C-terminal domains were swapped between Mlh1 and Mlh3 (abbreviated as PMM, MMP, PPM, PMP, MPP, MPM) and assessed for their ability to complement Mlh3’s major function in crossing over and minor function in MMR (Table 1). The endpoints for the domain swaps are presented in the *Materials and Methods* and Supplementary Figure S3B. None of the chimeras complemented MMR or meiotic crossover functions (Tables 1 and 2). Arguing against the possibility that the chimeric proteins were unstable was our finding that four Mlh3/Pms1 chimeric proteins (MMP, PPM, PMP, MPP) conferred mutation rates that were higher than seen for *mlh3Δ.* One explanation for this phenotype is that the *MMP*, *PPM*, *PMP*, and *MPP* chimeric genes expressed polypeptides that interfered with other MLH MMR pathways; for example, the chimeras could sequester Mlh1 from interacting with Pms1 in MMR [see examples in Shcherbakova and Kunkel (1999) and Smith *et al.* (2013)].

### Identifying residues critical for Mlh3 MMR and crossover functions

Our inability to identify domains in the MLH proteins that conferred functional specificity encouraged us to perform a more targeted approach. Because Mlh3 plays a minor role in MMR, and Pms1 does not act in dHJ resolution, we thought that our best opportunity to study the specificity of the MLH proteins was to make substitution mutations in *MLH3* that affected its MMR and meiotic crossing over functions. Such substitutions might also allow Mlh3 to partially replace Pms1 MMR functions (see below). We performed this analysis recognizing that the divergence of Mlh3 and Pms1 is an ancient event, and any changes in specificity would be informative as refinement of protein function would likely require large numbers of changes over a long evolutionary time scale.

We used multi-Harmony analysis as a targeted approach to identify residues in Mlh3 and Pms1 required for MMR function. Multi-Harmony uses multiple sequence alignments between subfamilies of proteins, homology models, and multi-Relief and sequence-Harmony algorithms to identify amino acids that may suggest functional specificity (*Materials and Methods*; Brandt *et al.* 2010). By aligning Mlh3 and Pms1 amino acid sequences from 34 fungal species (Supplementary Figure S5, A–D), residues were identified that are well conserved within each subfamily but differed between Mlh3 and Pms1 (Blocks 1–6). When mapped onto the 3D structure of Mlh1-Pms1 on PyMOL, clusters of amino acids were identified and labeled as “blocks.” The substitutions in each block are shown in the Mlh3 sequence with the Mlh3 residue shown first, the amino acid position in Mlh3, and the equivalent position in Pms1.

Block 1 maps to the ATP-binding domain; Block 2 to the Mlh1 interaction motif, and Blocks 3, 5, and 6, to the MLH endonuclease motif (Figure 2). Because of the extremely high conservation of some single residues in Mlh3 or Pms1, we also tested single amino acid substitutions with two (D500N, K502G) mapping to Block 2, two (R530K, R532N) to Block 3, and one (D678K) to Block 5. Lastly, a Block 4 was created based on previous studies showing that eukaryotic MutSα and MutLα complexes each interact with the DNA replication processivity clamp PCNA through a PIP Box defined as a six amino acid sequence consisting of Qxφ[L/I]xP, where φ is a hydrophobic residue and x is any amino acid (Kelman 1997; Pillon *et al.* 2011; Genschel *et al.* 2017; Supplementary Figure S3D). For MutSα, this interaction helps to tether at least a subset of MutSα complexes to the replication fork (Hombauer *et al.* 2011). For MutLα, interaction with PCNA through a PIP-motif present in the Pms1 endonuclease domain is critical to activate MutLα’s endonuclease activity (Pluciennik *et al.* 2010; Genschel *et al.* 2017). Mlh3 does not contain this motif in the corresponding position in its endonuclease domain, and Mlh1-Mlh3 endonuclease activity was not stimulated by PCNA under conditions where the Mlh1-Pms1 endonuclease is activated (Ranjha *et al.* 2014; Rogacheva *et al.* 2014; Manhart *et al.* 2017). However, Cannavo *et al.* (2020) recently identified candidate PIP motifs in both yeast Mlh1 and Mlh3 that when mutated conferred defects in meiotic crossing over and partially disrupted stimulation of Mlh1-Mlh3 endonuclease activity in the presence of PCNA, Exo1, and Msh4-Msh5. As described below, we introduced PIP motif substitutions into Mlh3 based on a homology model of the C-terminal domain of the Mlh1-Mlh3 complex (Supplementary Figure S3, C and D). Specifically, we introduced conserved PIP residues into a sequence (GTFVAR) in Mlh3 that corresponds to the location of the PIP motif in Pms1.

### Effect of Multi-Harmony substitutions on *MLH3* functions in MMR and crossing over

The *mlh3*-*Block1-6* substitutions were tested for complementing Mlh3 functions in MMR and meiotic crossing over (Table 1), and for their ability to complement Pms1 functions in MMR (Table 2). A brief summary of the phenotypes conferred by these phenotypes is shown below.

The *mlh3-Block1* (ATP binding) allele conferred an intermediate phenotype in the mutation rate assay (2.20-fold higher than *wild type*) but did not affect meiotic crossing over.

The *mlh3-Block2* (Mlh1 interaction) alleles conferred intermediate phenotypes in both the mutation rate assay (1.68- to 3.68-fold higher than *wild type*) and meiotic crossing over (19.1–33.1% tetratype, compared to 37.1% for *wild type*), but with one allele (*mlh3-K502G*) conferring a more severe defect in the meiotic crossover assay.

*mlh3-Block3, 5, 6* (Mlh3 endonuclease motifs) alleles conferred intermediate to severe phenotypes in both mutation rate and meiotic crossover assays, with some alleles conferring a more severe defect in one assay *vs* the other. It was interesting to see a more severe defect for the Block 3 *mlh3-R530K*, *R532N* double mutation compared to the single mutations (4.36-fold for the double mutation *vs* 2.31- and 1.21-fold for the single mutations in the mutation rate assay; 20.4% tetratype for the double mutation *vs* 25.3% and 29.5% tetratype for single mutations in the meiotic CO assay), indicating important roles for the two residues in Mlh3 function.

Two *mlh3-Block4* mutant alleles were created. *mlh3-Block4-PIP1* contains the yeast Pms1 PIP motif (**QKLIIP**) and *mlh3-Block4-PIP2* contains just the three critical consensus residues (**Q**TF**I**A**P**), with the other residues derived from Mlh3 (Supplementary Figure S3D). The *mlh3-Block4-PIP2* mutation complemented Mlh3 functions in both MMR and meiotic crossing over, whereas *mlh3-Block4-PIP1* conferred an intermediate defect in both assays (3.39-fold higher than *wild type* in the mutation rate assay, 30.9% tetratype in the CO assay). These observations encouraged us to perform the detailed analysis of the PIP motif described below.

Our observations confirmed that regions of high conservation specific to the Mlh3 family were critical for Mlh3 function; every Block substitution group conferred defects in MMR and meiotic crossing over, though with different phenotypic strengths. Also, the finding that some Block mutations differentially affected Mlh3 MMR and meiotic functions is consistent with the previous identification of Mlh3 separation of function mutations that may disrupt interactions with specific pathway components (Table 1; Al-Sweel *et al.* 2017; Claeys Bouuaert and Keeney 2017).

### Identifying Block mlh3 mutations that partially complement the MMR defect seen in pms1Δ strains

The studies presented in Table 1 encouraged us to test if the *mlh3-Block1-6* alleles could complement Pms1 MMR functions (Table 2). We first tested if the *MLH3_SK1_* gene used to make all of our multi-Harmony substitutions could complement, when expressed on an *ARS-CEN* single copy vector, the MMR functions of an S288c *mlh3Δ* strain (EAY4595). As shown in Table 1, this plasmid displayed full complementation, indicating that it was an appropriate gene construct for our analysis. We then asked if the suppression of the *mlh3-PIP1* and *PIP2* alleles expressed on an *ARS-CEN* vector could complement the *pms1Δ* mutator phenotype. As shown in Table 2, neither *mlh3* allele complemented the *pms1Δ* mutator phenotype.

The inability of the *mlh3-PIP1* and *-PIP2* alleles to complement the *pms1Δ* mutator phenotype encouraged us to express *mlh3* alleles through the *MLH3* promoter on a high copy *2*µ *LEU2* pRS derived vector. We were encouraged to overexpress the mutant proteins because an analysis of multiple protein abundance data sets (Ho *et al.* 2018) indicated that Mlh3 protein levels (median of 220 molecules per cell) are lower than those seen for Pms1 (median of 648 molecules per cell). Karim *et al.* (2013) determined in an S288c strain that such *LEU2 2µ* vectors were present at 14 copies/cell, and so the expression of *MLH3* in a *2µ LEU2* vector is predicted to yield Mlh3 molecule numbers per cell that are only a few-fold higher than the native Pms1 levels. Interestingly, mild overexpression of *MLH3* on a *2µ* vector in *mlh3Δ* conferred a mutation rate significantly higher than seen when expressing an empty *2µ* vector in *mlh3Δ* (81.8- *vs* 5.90-fold), but this rate was still significantly lower than seen in *pms1Δ* strains (6320-fold). This was not a surprise as we had shown previously that robust overexpression of *MLH3* through a *GAL10* promoter conferred mutation rates in a *wild-type* strain similar to that seen in *pms1Δ*, most likely by sequestering Mlh1 from interacting with Pms1 (Nishant *et al.* 2008). We reasoned that any improved function for an *mlh3* allele in *PMS1*-dependent MMR should overcome the elevated mutation rate associated with *MLH3* overexpression. However, it also seems possible that improved function could be due to both amino acid substitutions and changes in protein levels/stability that result from the substitutions.

**Figure 3:**
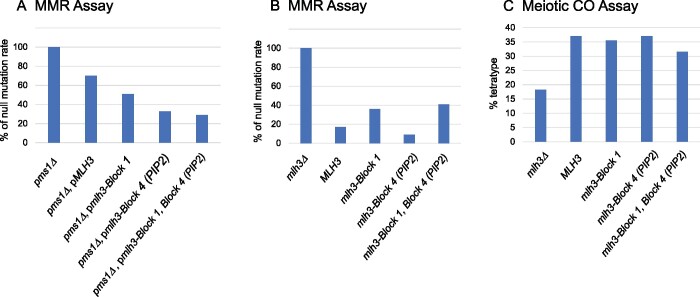
Block 1 and Block 4 *mlh3* mutations can alleviate the *pms1Δ* MMR defect. (A) Mutation rates of *mlh3-Block 1* and *mlh3-Block 4* single‐ and double‐mutant alleles expressed on *2µ* plasmids in *pms1Δ* strains were determined in the *lys2-A_14_* assay as described in *Materials and Methods*. Rates are shown as a percentage of the corresponding *pms1Δ*. (B) Mutation rates of strains containing *mlh3* constructs integrated at the *MLH3* locus in *PMS1* strains. Rates are shown as a percentage of an *mlh3Δ* strain. (C) Meiotic crossover phenotypes, expressed as % tetratype, for diploid strains containing *mlh3* constructs integrated at the *MLH3* locus. For (A), MMR rates were normalized from data presented in Table 2, with the lines from the table where the data were obtained indicated with *PMS1*, *ARS-CEN* corresponding to line 1. Data were obtained from *pms1Δ* strains containing an empty *2µ* vector-line 4 (set to 100%), p*MLH3*-line 48, p*mlh3-Block 1-*line 10, p*mlh3-Block 4 (PIP2)-*line 23, and p*mlh3 Block 1, Block 4 (PIP2)-*line 37. For (B), MMR rates were normalized from data presented in Table 1 (lines indicated, with *MLH3* corresponding to line 1) and obtained as follows: *mlh3Δ*-line 2 (set to 100%), *MLH3*-line 1, *mlh3-Block 1*-line 13, *mlh3-Block 4 (PIP2)-*line 24, and *mlh3-Block 1, Block 4 (PIP2)-*line 32. For (C), CO data were obtained from data presented in Table 1 (lines indicated) as follows: *mlh3Δ-*line 2, *MLH3*-line 1, *mlh3-Block 1*-line 13, *mlh3-Block 4 (PIP2*)-line 24, and *mlh3-Block 1, Block 4 (PIP2)*-line 32.

As shown in Table 2 and Figure 3, *mlh3*-*Block1* and *mlh3-Block4-PIP2* alleles expressed on *2µ* vectors conferred modest suppression of the *pms1Δ* MMR defect (2- to 3.5-fold lower mutation rates), whereas *mlh3*-*Block2, 3, 5, 6* alleles and the six chimera constructs did not. The failure of the *mlh3-Block4-PIP1* allele, which contained the complete PIP consensus, to suppress the *pms1Δ* MMR defect suggested that the suppression seen with *mlh3-Block4-PIP2* was not due to Mlh3 being able to interact with PCNA in MMR. To better understand if a canonical PIP motif was important for this improved complementation, we made a total of nine substitutions in the Mlh3 GTFVAR sequence (Supplementary Figure S3D). The finding that *mlh3-PIP5* (QTFIAR) and *mlh3-PIP9* (QTFVAR*)* strains (both missing leucine/isoleucine and proline residue conservation at positions 4 and 6, respectively) maintained modest complementation (2.1- to 2.7-fold lower mutation rates) whereas the *mlh3-PIP1* strain did not, provided the most relevant data suggesting that the improved MMR functions in the *mlh3-PIP2* strain were unlikely due to the creation of a PCNA interaction. The other substitutions, which were focused on introducing one, two, or three conserved PIP residues into the Mlh3 GTFVAR sequence were not especially informative because none of them improved *MLH3* MMR functions (*mlh3-PIP3, 4, 6, 7, 8*). Together, these findings suggest that the addition of a functional PIP motif was unlikely to explain the improved MMR phenotype seen in *mlh3-Block4-PIP2, -PIP3*, and *-PIP5*. In addition, they do not address whether the mlh3-PIP proteins are directly interacting with PCNA, or if a structural change induced by the insertion of this motif alters its endonuclease activity *in vivo* to be more similar to that seen with Mlh1-Pms1.

The above observations encouraged us to determine if the modest complementation seen for the *mlh3-Block1* and *mlh3-Block4-PIP2 alleles* could be enhanced or maintained in combination with other *mlh3* alleles. As shown in Table 2 and Figure 3, the *mlh3-Block1, Block4-PIP2* double mutation complemented the *pms1Δ* MMR defect as well, if not slightly better than the *mlh3-Block1* or *mlh3-Block4-PIP2* mutations alone. Double-mutant combinations that included *mlh3-Block4-PIP2* and alleles that did not, or only weakly affected *MLH3* function (*D678K*, *R530K*, *R532N* singles) displayed mutation rates similar to the *mlh3-Block4-PIP2* mutant alone. However, other hypomorph *mlh3* alleles (*D500N*, *K502G* singles), disrupted the ability of *mlh3-Block4-PIP2* to suppress the *pms1Δ* MMR defect. It was interesting to see that the severe *mlh3-R530K, R532N* and *mlh3-Block6* mutations disrupted *mlh3-Block4-PIP2* complementation, consistent with the idea that Block mutations that could not complement *mlh3Δ* and *pms1Δ* functions were deleterious for all MLH functions. Together, these observations indicate that multi-Harmony is a valuable approach to identify residues outside of consensus motifs that are critical for specificity of subsets of protein family members. The finding that substitutions located in both the ATP-binding and endonuclease domains of Mlh3 and Pms1 could alter Mlh3 specificity supports ERC analysis indicating that multiple domains across the MLH proteins are co-evolving to provide specificity. It is also of interest because the N-terminal domains of Mlh1 and Pms1 were shown to independently bind to double-stranded and single-stranded DNA (Hall *et al.* 2003; see also Claeys Bouuaert and Keeney, 2017), suggesting that both the N- and C-terminal domains of MLH proteins contribute to interactions with DNA that could provide specificity for the different MLH pathways.

## Discussion

We leveraged phylogenetic, multi-Harmony, and molecular genetic analyses to alter the specificity of Mlh3 to modestly complement Pms1 MMR functions. We were surprised by the partial complementation of Pms1 MMR functions because the functional advantage conferred by making a limited number of mutations in proteins that differ by hundreds of amino acids would likely be difficult to detect. The altered specificity seen in *mlh3* alleles was further confirmed by combining mutant combinations. Overall, combining beneficial alleles maintained or slightly improved altered specificity, whereas combining a neutral allele with a beneficial one did not affect outcome, and combining a deleterious allele with a beneficial one disrupted the altered specificity. These data, combined with chimera and ERC analyses, are consistent with the three domains of the MLH proteins being interdependent, having coevolved for millions of years, and contributing to the distinct specificities in MMR and meiotic crossing over seen for the Pms1 and Mlh3 proteins, respectively.

Two models were presented in the *Introduction* to explain how novel protein functions evolved after gene duplication. We find it intriguing that two *mlh3* alleles (*Block1* and *Block4*) displayed partial complementation of Pms1 MMR functions yet maintained Mlh3 meiotic crossover functions (Figure 3), suggesting that the specialization of the MLH functions could be partially reversed. Such an observation is consistent with an ancestral MLH protein having displayed both MMR and meiotic functions that became specialized following a gene duplication event (model presented by Hughes 1994). Our observations could also be explained by a DDC model (Force *et al.* 1999) where duplication of *MLH* genes resolves conflicts seen for an ancestral gene that performs both functions. However, our findings suggesting that the specialization of Mlh3 functions could be partially reversed appears less consistent with the DDC model, which proposes that neutral degenerative mutations occur in each duplicated gene that complement each other.

Our work complements a recent study, which examined the evolution of factors that act in vegetative and meiotic functions. In baker’s yeast, the Rad51 and Dmc1 strand exchange proteins, which share ∼50% amino acid identity and are thought to evolve from a single-gene duplication of an ancestral recombination protein, play critical roles in promoting homologous recombination [reviewed in Furman *et al.* (2021)]. Both proteins are able to catalyze the invasion of a 3ʹ single-stranded end into a homologous duplex template to form postsynaptic complexes in three nucleotide steps (Brown and Bishop 2014; Lee *et al.* 2015; Chan *et al.* 2019; Steinfeld *et al.* 2019). Most eukaryotes contain a Rad51 protein that is expressed in both mitotic and meiotic cell cycles, and a Dmc1 protein which is meiosis-specific and unlike Rad51, can stabilize heteroduplex DNA with mismatch containing base triplets. In meiosis, Dmc1 and Rad51 play somewhat complementary roles, with Dmc1 catalyzing interhomolog recombination, and Rad51 promoting the assembly of the Dmc1 presynaptic filament (Neale and Keeney 2006; Lao *et al.* 2013; Brown and Bishop 2014). Analogous to our work, Steinfeld *et al.* (2019) examined residues that are conserved within the Rad51 or Dmc1 lineages but differ between them. They then made Rad51/Dmc1 chimeras and identified a set of three amino acid substitutions in the L1 DNA-binding loop of these proteins that conferred differences in how the two proteins stabilized recombination intermediates bearing base triplets with mismatches; only Dmc1 can stabilize such intermediates. While such changes in specificity were observed biochemically, they did not result in the formation of functional proteins *in vivo* as was seen for the modest complementation that we observed for the *mlh3* Block 1 and 4 alleles. Steinfeld *et al.* (2019) speculated that swapping this motif “may hinder some downstream step in the HR pathway.” Furthermore, they identified 19 lineage-specific amino acids in other regions of the two recombinases that they speculated were important for Rad51 and Dmc1-specific protein–protein contacts. Thus, this study and ours show the challenges in achieving full complementation in proteins that have become highly specialized and significantly diverged from ancestral proteins.

Our study raises the question of whether it would be possible to identify *mlh3* alleles with significantly improved functions in the *PMS1* MMR pathway. When we started this work, we recognized that making *mlh3* alleles with altered specificity would be challenging because Mlh3 and Pms1 work with a multitude of other proteins in their respective meiotic and MMR pathways [reviewed in Furman *et al.* (2021)]. We attempted to alter the specificity of *MLH3* by only making a small number of amino acids changes. More extensive changes could be made, for example, by using advanced computational modeling approaches. Such methods could be used to identify an ancestral state of Mlh3 capable of acting more efficiently in MMR (see reviews by Thornton 2004; Gumulya and Gillam 2017). Alternatively, one could perform an extensive mutagenesis to identify *mlh3* alleles that counter a strong selection such as conferring viability in *pms1Δ pol3-01* haploids that are inviable due to high mutational load (Morrison *et al.* 1993; Argueso *et al.* 2002). An analogous experimental evolution approach was performed in budding yeast by Hsieh *et al.* (2020), who identified mutations in *REC8* and in other genes that conferred viability in vegetative growth when the meiotic kleisin Rec8 was expressed in the absence of the vegetative paralog Scc1.

## Notes

These authors are co-first authors.
